# Case report: Dupilumab treatment improved type 2 disorders in a patient with IPEX syndrome diagnosis

**DOI:** 10.3389/fimmu.2022.995304

**Published:** 2023-01-11

**Authors:** C. Caruso, L. Laterza, C. R. Settanni, S. Colantuono, C. Di Mario, B. Tolusso, F. Castrì, E. Gremese, F. Scaldaferri, A. Armuzzi, C. De Simone, K. Peris, A. Chiricozzi, A. Gasbarrini

**Affiliations:** ^1^ Dipartimento di Scienze Mediche e Chirurgiche, Fondazione Policlinico Universitario A. Gemelli, IRCCS, Rome, Italy; ^2^ UOC di Medicina Interna e Gastroenterologia, Dipartimento di Scienze Mediche e Chirurgiche, Fondazione Policlinico Universitario A. Gemelli IRCCS, Rome, Italy; ^3^ Immunology Core Facility, Gemelli Science Technological Park (GSTeP), Fondazione Policlinico Universitario A. Gemelli IRCCS, Rome, Italy; ^4^ Division of Clinical Immunology, A. Gemelli University Hospital Foundation IRCCS, Rome, Italy; ^5^ Dipartimento di Anatomia Patologica, Fondazione Policlinico A. Gemelli, IRCCS, Rome, Italy; ^6^ Istituto di Patologia Speciale Medica, Università Cattolica del Sacro Cuore, Rome, Italy; ^7^ Faculty of Medicine and Surgery, Catholic University of the Sacred Heart, Rome, Italy; ^8^ IBD Center, IRCCS Humanitas Research Hospital, Rozzano, Milan, Italy; ^9^ Department of Biomedical Sciences, Humanitas University, Pieve Emanuele, Milan, Italy; ^10^ Institute of Dermatology, Università Cattolica del Sacro Cuore, Rome, Fondazione Policlinico Universitario A. Gemelli IRCCS, Rome, Italy; ^11^ Faculty of Internal Medicine, Catholic University of the Sacred Heart, Rome, Italy; ^12^ Internal Medicine and Gastroenterology Unit, Fondazione Policlinico Universitario A. Gemelli IRCCS, Rome, Italy

**Keywords:** cytokines, atopic dermatis, type2, biomarker, dupilumab, IPEX (immune dysregulation, polyendocrinopathy, X-linked)

## Abstract

We described a case of IPEX syndrome successfully controlled with dupilumab, an anti-IL4 receptor alpha subunit inhibitor. IPEX syndrome is a rare and generally fatal genetic disorder characterized by immune dysregulation, polyendocrinopathy and enteropathy, mostly diagnosed in early childhood. Nonetheless, cases reported in the last 20 years demonstrated that IPEX clinical spectrum encompasses more than the classical triad of early-onset intractable diarrhea, type 1 diabetes and eczema. Atypical cases of IPEX include patients with late-onset of symptoms, single-organ involvement, mild disease phenotypes or rare clinical features. A 21-year-old caucasian man presented with immune dysregulation (hypereosinophilia and elevated IgE), protein-losing enteropathy, polyendocrinopathy (thyroiditis, osteoporosis, delayed puberty), weight loss, eczema manifestations and celiac disease. IPEX syndrome was diagnosed because of the presence of a hemizygous mutation in FOXP3 gene (c.543C>T (p.S181S) in the exon 5). During the course of the disease, the patient developed erosive proctitis, pyoderma gangrenosum, and erythema nodosum. Symptoms improved only after enteral and parenteral corticosteroid therapy and the patient soon developed steroid-dependence. Notwithstanding various therapies including azathioprine, sirolimus, tacrolimus, adalimumab, vedolizumab, the patient failed to achieve a good control of symptoms without steroids. Almost exclusive enteral nutrition with a hypoallergenic, milk-protein free, amino acid-based food for special medical purposes. He continued to lose weight (BMI 14.5 kg/m2) with a consequent high limitation of physical activity and a progressive worsening of the quality of life. In consideration of the poor response to conventional immunosuppressants and the presence of type 2 inflammatory manifestations, treatment with dupilumab at an initial dose of 600 mg, followed by a maintenance dose of 300 mg every other week, according to atopic dermatitis labeled dose, was started and combined to oral budesonide 6 mg/day and 6-mercaptopurine 75 mg/day. The patient experienced a rapid improvement in bowel and skin symptoms, leading to a progressive tapering of steroids. By our knowledge, this is the first report of IPEX syndrome successfully treated by antiIL-4/IL-13 therapy. In this case dupilumab demonstrated to be an effective, safe and steroid-sparing option.

## Introduction

IPEX is a primary immunodeficiency caused by mutations in FOXP3 gene, whose treatment is still challenging since new clinical options have remained elusive. Dupilumab has been proved effective and safe in the treatment of type 2 disorders associated with IPEX in this patient.

IPEX syndrome is a rare and generally fatal genetic disorder characterized by immune dysregulation, polyendocrinopathy and enteropathy, mostly diagnosed in early childhood.

Despite the clinical heterogeneity, the unifying feature of IPEX is mutation of the FOXP3 gene, which encodes a transcription factor essential for maintenance of thymus-derived regulatory T cells (Tregs) (1).

The impaired functioning of Treg cells makes the patient prone to the development of immune mediated inflammatory diseases, such as thyroiditis and type I diabetes. Usually, intractable diarrhea can lead to malabsorption, maldevelopment and weight loss (2). In the majority of cases, skin manifestations appears as eczematous patches and irritated skin, though psoriasis-like lesions, urticaria, alopecia universalis, bullous pemphigoid, and trachyonychia have also been described. Currently, immunosuppressive drugs (IS) and bone hematopoietic stem cell transplantation (HSCT) are the main treatments for IPEX syndrome, as well as long-term supportive care, including parenteral nutrition (3).

Nonetheless, cases reported in the last 20 years demonstrated that IPEX clinical spectrum encompasses more than the classical triad of early-onset intractable diarrhea, type 1 diabetes and eczema. Atypical cases of IPEX include patients with late-onset of symptoms (early childhood or adolescence), single-organ involvement, mild disease phenotypes or rare clinical features (4).

**Figure 1:**
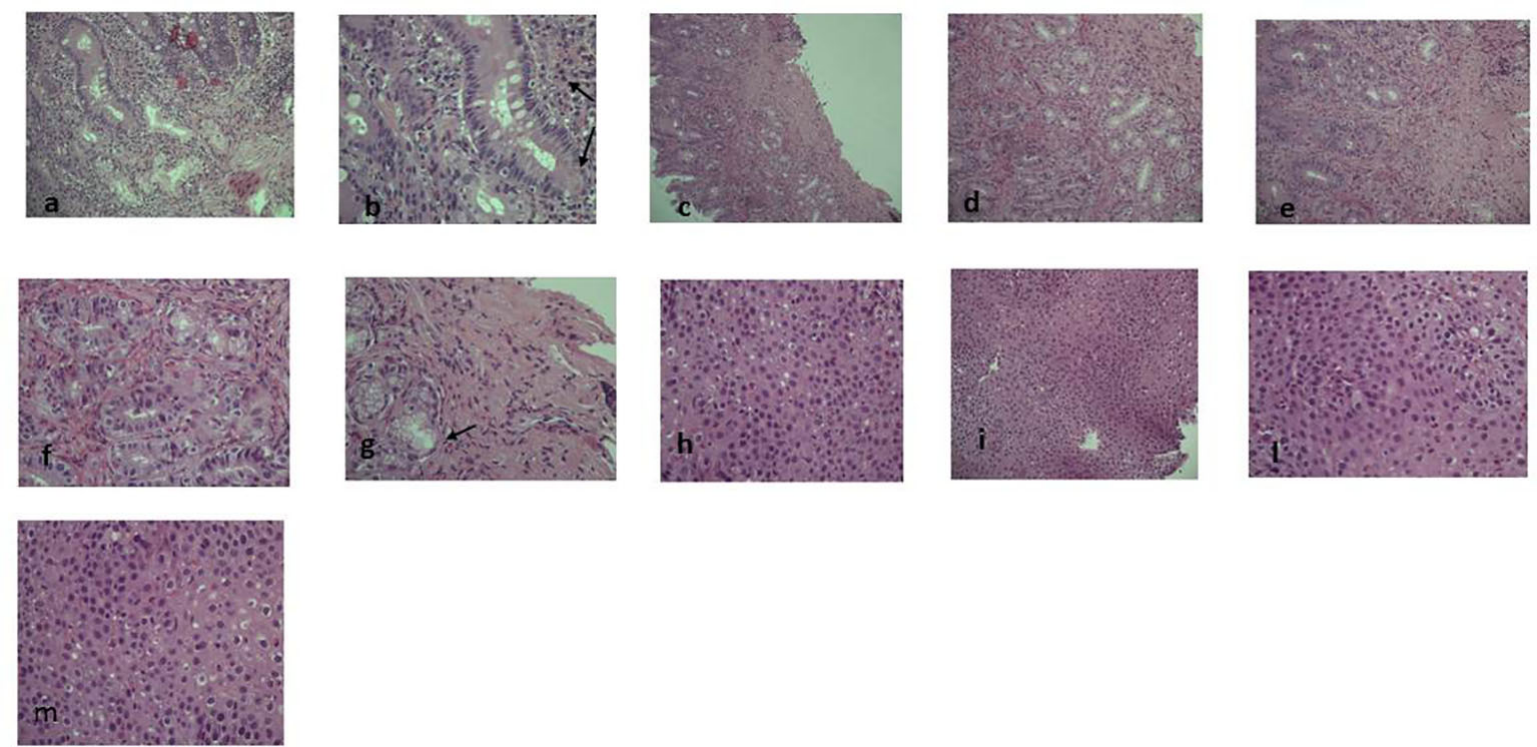
Pre-dupilumab histological findings. The figure shows the pre-therapy gastrointestinal eosinophilic infiltrate at different levels: **(A–M)(A)** (EE 10x) and **(B)** (EE 20x): glandular and lamina propria eosinophilic infiltrate in duodenal mucosa (arrow). **(C)** (EE 5x), **(D)** (EE 10x), **(E)** (EE 20x), **(F)** and **(G)** (EE 40x): gastric eosinophilic infiltrate (>50xHPF) either in glands and in lamina propria and in muscularis mucosae with a micro-erosive intraglandular pattern (arrow). **(H, L, M)** (EE 40x) and **(I)** (EE 10x): eosinophilic intraepithelial infiltrate (35-40xHPF).

In this report we described a case of IPEX syndrome successfully controlled with dupilumab, an anti-IL4 receptor alpha subunit inhibitor, which blocks both IL-4 and IL-13 signals (5). A 21-year-old caucasian man presented with immune dysregulation (hypereosinophilia and elevated IgE), protein-losing enteropathy, polyendocrinopathy (thyroiditis, osteoporosis, delayed puberty), weight loss, eczema manifestations and celiac disease. He started presenting gastrointestinal symptoms by the age of 3, particularly loss of appetite, nausea, heartburn, vomiting and recurrent diarrhea, thus he was diagnosed with eosinophilic gastroenteritis by biopsy (**Figure 1**) and subsequently identified as IPEX syndrome because of the presence of a hemizygous mutation in FOXP3 gene (c.543C>T (p.S181S) in the exon 5). Only a brother has been tested for the mutation and he also carried it, although asymptomatic at that time (6). Anti-tissue transglutaminase and anti-deaminated gliadin antibodies were positive in the patient.

During the course of the disease, the patient developed erosive proctitis, pyoderma gangrenosum, erythema nodosum. The patient also developed atopic disorders including allergic rhinitis and atopic dermatitis.

Symptoms improved only after enteral and parenteral corticosteroid therapy and the patient soon developed steroid-dependence. Thereby, after many years of prednisone, a continuous therapy with high-dose topical steroids (oral budesonide 6 to 9 mg/day) was continuously administered, in order to reduce steroid-related systemic effects and maintain control of symptoms. After a first allergist evaluation, diet was severely restricted, as many foods were eliminated only because of the presence of specific serum IgE, albeit no history of any adverse reaction to food was reported. The patient used to have a milk-, egg- corn-, fish-, meat-, and gluten-free diet. Because of malabsorption, restricted dietary regimen and steroid therapy, the patient developed delayed puberty (at 16 years) and osteoporosis. Notwithstanding various therapies including azathioprine, sirolimus, tacrolimus, adalimumab, vedolizumab, the patient failed to achieve a good control of symptoms without steroids. Thus, during treatment with 6-mercaptopurine and budesonide, he developed severe nausea, heartburn, non-bloody diarrhea (10 bowel movement per day) and loss of appetite. Further diet restriction was established, starting an almost exclusive enteral nutrition with a hypoallergenic, milk-protein free, amino acid-based food for special medical purposes. He continued to lose weight (BMI 14.5 kg/m^2^) with a consequent high limitation of physical activity and a progressive worsening of the quality of life.

Laboratory exams showed hypertransaminasemia, high IgE levels (715 UI/mL) and hypereosinophilia (2.08 x 10^9/L). Moreover, before starting dupilumab treatment, IL-4 serum levels were 0.92 pg/ml [Enzyme-Linked Immunosorbent Assay (ELISA) method, Bio-Techne, UK] and IL-13 serum levels were undetectable (ELISA method, Bio-Techne, UK) (7). Dermatologic inspection reported eczematous lesions localized on flexural areas, lower limbs, wrists, and arms. SCORAD (SCORing Atopic Dermatitis) was 32; BSA (Body Surface Area) 8; itch NRS scale: 6; sleep NRS scale: 4. Histology obtained from endoscopic biopsies showed diffuse gastrointestinal eosinophilic infiltration (**Figure 1**).

In consideration of the poor response to conventional immunosuppressants and the presence of type 2 inflammatory manifestations, treatment with dupilumab at an initial dose of 600 mg, administered subcutaneously, followed by a maintenance dose of 300 mg every other week, according to atopic dermatitis labeled dose (8), was started and combined to oral budesonide 6 mg/day and 6-mercaptopurine 75 mg/day. The patient experienced a rapid improvement in bowel and skin symptoms, leading to a progressive tapering of steroids. Therapy with 6-mercaptopurine was discontinued after 12 months of dupilumab therapy. During the first year of treatment, meat, fish, milk, cheese and egg (cooked yolk) were gradually added to the diet and well tolerated. In particular, in this patient, sensitization to wheat was probably due to cross-reactivity, that is common in the grass-allergic children, in particular to Phlp 12 allergen, and also associated with allergy to staple foods other than wheat (9). Therefore, according to the celiac disease diagnosis, a gluten-free diet was continued, but the patient gradually started to reintroduce corn and other cereals. At 12 months follow-up, IL-4 serum levels decreased to 0.15 pg/ml and, unexpectedly, IL-13 serum levels raised up to 9.33 pg/ml. Various studies focused on the suppression of circulating levels of most type 2 inflammatory biomarkers in different type 2 diseases treated with dupilumab (10). Although IL-4 and IL-13 indisputably play central roles in type 2 inflammation, varying effects of these cytokines, resulting in differences in the effector cells involved in the pathogenesis of each disease, may occur. The disconnection of IL-4 and IL-13 levels in this patient remains an unsolved issue, probably attributable to a more complex immune dysregulation that characterizes this syndrome.

**Figure 2:**
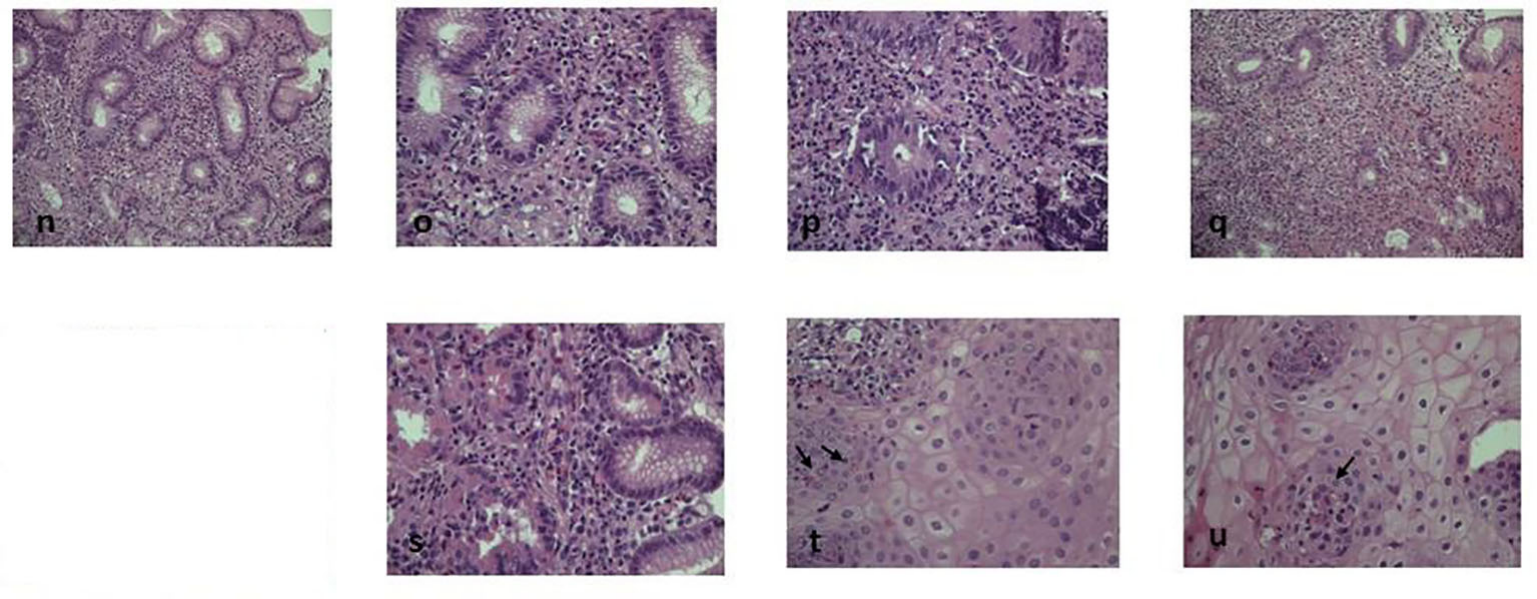
Post-dupilumab histological findings. Eosinophilic infiltrate strongly decreased after biological therapy. Figure shows 9 months post-therapy histology at different levels: n-u: **(N)** (EE20x), **(O)** and **(P)** (EE 40x) duodenal and **(Q)** (EE20x), **(R)** (EE20x) and **(S)** (EE40x) gastric strongly decreased granulocitic infiltrate (<10xHPF without aggressive infiltrating pattern) **(T, U)** (EE 40x): less than 4-5 intraepithelial esophageal eosinophils xHPF can be detected (arrows).

After 18 months of follow-up, the patient achieved an almost complete remission of skin and intestinal disease, obtaining a weight gain of 7 kg (BMI 18.3 kg/m^2^) and a progressive reduction of budesonide to complete withdrawal, with a favorable impact on the quality of life and restarting of the physical activity. Laboratory exams showed a normalization of liver abnormalities and eosinophils (0.8 x 10^9/L). Moreover, endoscopic procedures, repeated after 9 months, showed a global depletion of eosinophils in the tissues (**Figure 2**). The organ involvement (OI) scoring system represents the number of organs or systems impaired by the autoimmune damage or by secondary complications (11). Intractable diarrhea, malnutrition, liver dysfunction, respiratory impairment, kidney dysfunction are assessed and scored by caring physicians according to standard of care definitions (1 point for each manifestation). The effect of IS is considered “beneficial” when the physician observes a decrease (partial benefit) or complete disappearance (benefit) of signs and symptoms of the disease. At the diagnosis, the presence of diarrhea, malnutrition, liver and respiratory involvement scored 4 for this patient (range 0-5). The OI score showed a reduction from 4 to 1 from the beginning of the treatment. Notably, no adverse event was reported during the follow-up.

## Concluding remarks

By our knowledge, this is the first report of IPEX syndrome successfully treated by antiIL-4/IL-13 therapy. The most recent literature showed that only half IPEX syndrome are caused by a pathogenic mutation in the FOXP3 gene and often IPEX-like syndrome are indistinguishable from them. The complexity of interactions of FOXP3 with other genes and its epigenetic regulation can be responsible for phenotype variability, rather than FOXP3 variants themselves (6). This patient surely showed atypical features of IPEX, i.e. late-onset of symptoms, “mild” phenotype, never requiring a stem cell transplant and unexpected prolonged survival. Nevertheless, severe clinical manifestations and a very poor quality of life imposed an effective treatment and in this case dupilumab demonstrated to be effective and safe in the treatment of type 2 disorders associated with IPEX. Moreover, it represented a useful steroid-sparing option in this case.

## Data availability statement

The datasets presented in this study can be found in online repositories. The names of the repository/repositories and accession number(s) can be found in the article/supplementary material.

## Ethics statement

Ethical review and approval was not required for the study on human participants in accordance with the local legislation and institutional requirements. The patients/participants provided their written informed consent to participate in this study. Written informed consent was obtained from the individual(s) for the publication of any potentially identifiable images or data included in this article.

## Author contributions

CC and LL wrote the paper, SC reviewed the literature and reviewed the paper, the rest of author’s they cooperated in the submission. All authors listed have made a substantial, direct, and intellectual contribution to the work and approved it for publication.
